# Feasibility of primary sternal plating for morbidly obese patients after cardiac surgery

**DOI:** 10.1186/s13019-019-0841-y

**Published:** 2019-01-28

**Authors:** Joshua M. Liao, Patrick Chan, Lorraine Cornwell, Peter I. Tsai, Joseph H. Joo, Faisal G. Bakaeen, James D. Luketich, Danny Chu

**Affiliations:** 1 Department of Medicine, University of Washington, Seattle, WA USA; 2 Department of Cardiothoracic Surgery, University of Pittsburgh School of Medicine, Pittsburgh, PA USA; 3 Department of Surgery, University of Hawaii, Honolulu, HI USA; 4 Division of Cardiothoracic Surgery, Michael E. DeBakey Veterans Affairs Medical Center, Houston, TX USA; 5 College of Medicine, Texas A&M University, Bryan, TX USA; 6 Department of Thoracic and Cardiovascular Surgery, Cleveland Clinic, Cleveland, OH USA; 7 University of Pittsburgh Medical Center Heart & Vascular Institute, 200 Lothrop Street, C-700, Pittsburgh, PA 15213 USA

**Keywords:** Obesity, Sternal wound dehiscence, Sternal plating, Cardiac surgery

## Abstract

**Background:**

Morbidly obese patients (body mass index [BMI] ≥ 35 kg/m^2^) who undergo cardiac surgery involving median sternotomy have a higher-than-normal risk of sternal dehiscence. To explore a potential solution to this problem, we examined the utility of transverse sternal plating for primary sternal closure in morbidly obese cardiac surgical patients.

**Methods:**

We retrospectively reviewed data from cardiac surgical patients who underwent single primary xiphoid transverse titanium plate reinforcement for primary sternal closure from August 2009 to July 2010 (*n* = 8), and we compared their outcomes with those of patients with BMI ≥35 kg/m^2^ who underwent cardiac surgery without sternal plate reinforcement from April 2008 to July 2009 (*n* = 14). All cases were performed by the same surgeon.

**Results:**

The 2 groups of patients had similar demographics and comorbidities (*P* > 0.05 for all). All patients with sternal plate reinforcement reported sternal stability at last follow-up (at a median of 27 months postoperatively; range, 8.4–49.3 months), whereas 1 patient (7.1%) who underwent standard closure developed sterile sternal dehiscence (*P* = 0.4). Postoperative patient-controlled analgesia (PCA) morphine usage was significantly higher for patients without sternal plate reinforcement than for patients who had sternal plate reinforcement (3.6 mg/h vs 1.3 mg/h, *P* = 0.008). No patient in the sternal plate group had wound seroma or perioperative complications attributable to sternal closure technique.

**Conclusion:**

Single xiphoid transverse plate reinforcement for primary sternal closure is a feasible option for morbidly obese patients, who are otherwise at high risk of developing sternal dehiscence. Using this technique may decrease postoperative narcotics usage.

**Ultramini abstract:**

Morbidly obese patients (body mass index ≥35 kg/m^2^) have a higher-than-normal risk of sternal dehiscence after cardiac surgery. In a pilot study, we found that those who underwent transverse sternal plating (*n* = 8) had no sternal dehiscence and required less postoperative analgesia than patients who underwent standard wire closure (*n* = 14).

## Introduction

Obesity is an alarming and rapidly growing problem among Western populations. It is associated with increased healthcare costs and with morbidity and mortality from a myriad of conditions, including coronary artery disease (CAD) [1, 2]. Obesity increases patients’ risk of developing cardiovascular risk factors such as diabetes, hypertension, insulin resistance, and dyslipidemia, and it acts through those risk factors to cause cardiac complications [3]. Approximately two thirds of patients who develop myocardial infarction have a body mass index (BMI) of 25 kg/m^2^ or greater [4], and with such a strong association between obesity and CAD, the number of obese patients requiring surgical intervention will only increase.

The impact of obesity on CAD-associated mortality, however, is not limited to the development of disease. In patients who undergo coronary artery bypass grafting (CABG), the development of postoperative deep sternal wound infections (DSWI) is rare but is associated with high mortality rates: some studies report rates between 14 and 47% in patients with DSWI, compared with approximately 2 to 5% in uninfected controls [5]. However, because of issues with body habitus, sternal instability, and nonunion, CABG patients with above-average BMI (especially those with BMI ≥ 40 kg/m^2^) are at a greater risk than other CABG patients for disruption of their median sternotomy and development of sternal dehiscence and postoperative DSWI [6].

Primary wire closure has traditionally been the preferred method of closure for median sternotomy incisions, and more than 40 different closure techniques have been suggested for optimizing sternal stability [7, 8]. Several studies have investigated their effectiveness, and others have examined the biomechanical properties of the different methods [9–12]. Additionally, studies have shown that in patients with wound instability, dehiscence, or DSWI, re-entry into the chest and rewiring of sternotomy incisions increase the risk of perioperative mortality [13, 14]. As such, rigid fixation with sternal plating has recently begun to be used for sternal closure in such patients. Early clinical, cadaveric, and biomechanical studies show sternal plating to be a reliable method for stabilizing the sternum in complicated cases [15–17]. For example, Fawzy et al., in a human cadaveric model, showed that it required signifincantly increased intrathoracic pressure to cause a 2.0 mm separation in wires with one sternal plate reinforcement vs. wires alone. They concluded that adding a single sternal plate to primary closure improves the strength of the sternum [15]. Additionally, Snyder et al. showed that postoperative length of stay was significantly shorter in patients who had sternotomy closure with sternal plates [17].

Although transverse sternal plating has been used successfully in secondary sternal reconstructions [13], there are limited data on its use for primary sternal closure in morbidly obese patients. The aim of the present study was to evaluate the short-term outcomes of primary sternal plating for sternotomy closure in morbidly obese patients who underwent cardiac surgery.

## Materials and methods

The study was granted waiver of consent and approved by the institutional review boards at the Michael E. DeBakey Veterans Affairs Medical Center (MEDVAMC) and Baylor College of Medicine, Houston, Texas. Data were obtained from the MEDVAMC’s Continuous Improvement in Cardiac Surgery Program (CICSP) database and from a retrospective review of the computerized patient record system. The CICSP database, organized by the Department of Veterans Affairs (VA) to provide continuous assessment and improvement of quality of care for all patients who undergo cardiac surgery in VA hospitals, contains comprehensive data (on > 140 variables, including demographic, clinical, outcome, and resource variables) collected prospectively at prespecified time points from all cardiac surgical patients in the VA Health Care System [18]. Data in the CICSP database were collected by a research nurse who reviewed each patient’s computerized medical record.

**Fig. 1:**
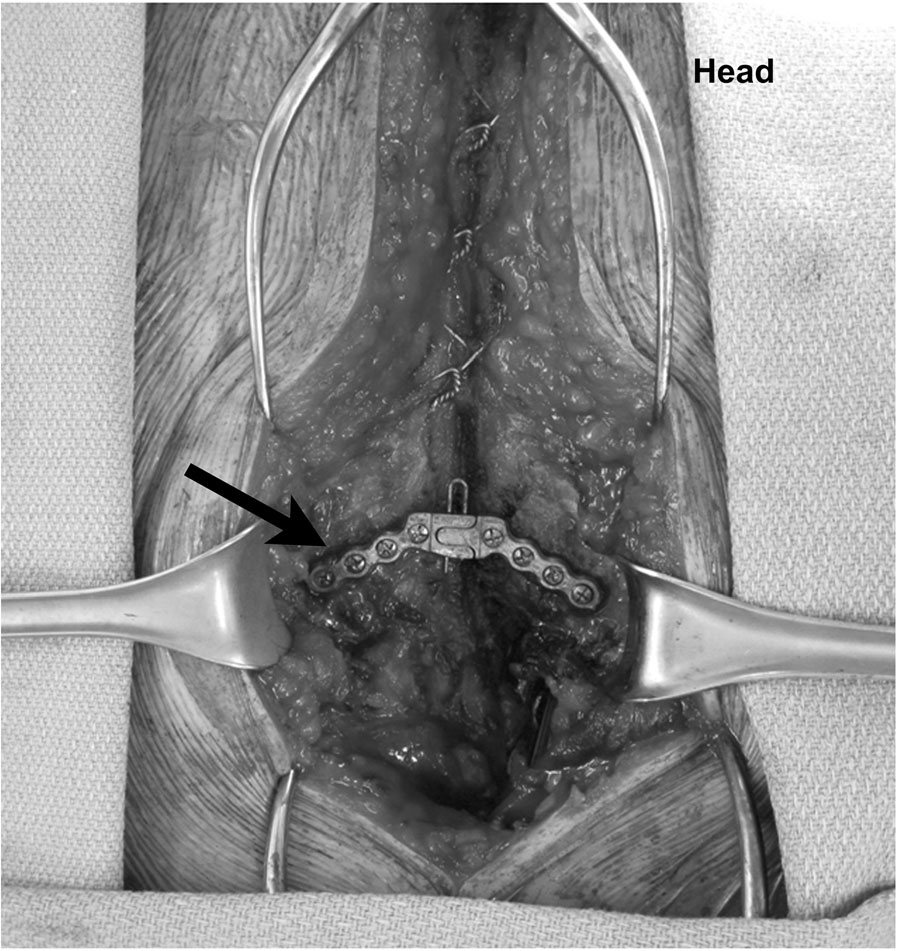
Intraoperative photograph of a Synthes titanium plate (arrow) after placement in the most inferior aspect of the sternum

We retrospectively reviewed data from all patients (each with BMI ≥ 35 kg/m^2^) who had sternal plate xiphoid reinforcement for primary sternotomy closure from August 2009 to July 2010 (*n* = 14) and compared their outcomes with those of patients with BMI ≥ 35 kg/m^2^ who underwent sternotomy closure without sternal plate reinforcement from April 2008 to July 2009 (n = 14). All operations were performed by the same surgeon (DC). All patients underwent standard closure with stainless-steel wires in an interrupted figure-of-eight fashion. Sternal plate reinforcement consisted of a single titanium plate secured with screws in the most inferior aspect of the sternum (Fig. 1). The decision to use sternal plate reinforcement was made at the discretion of the surgeon and was primarily based on the patient’s body habitus and/or the quality of sternal bone. Ten of the sternal plates used were supplied by the Synthes Titanium Sternal Fixation System (Synthes CMF, Paoli, PA); the remaining 4 plates were supplied by the Lorenz Sternalock System (Biomet Microfixation, Jacksonville, FL).

To reduce hardware bias, we only included patients who had Synthes plate reinforcement (*n* = 10) in our sternal-plate group. Because postoperative narcotics use was one of our outcome measures, we excluded patients who were ventilator-dependent for > 48 h due to increased pain or sedative medications while on the ventilator, patients who were taking narcotics or gabapentin preoperatively, patients who had postoperative stroke, and patients whose postoperative morphine usage record was incomplete or absent. Two patients were excluded from the sternal-plate group for these reasons. Thus, our final study cohort consisted of 8 patients who underwent sternal plate reinforcement and the 14 control patients who underwent standard wire closure.

### Study end point and statistical analysis

The primary end point of the study was the incidence of sternal dehiscence and mediastinitis as defined by CICSP [18]. The secondary end points were 30-day mortality, postoperative length of stay, and immediate postoperative patient-controlled analgesia (PCA) morphine usage. Sternal dehiscence was determined clinically and confirmed by chest computed tomography (CT) scan. Postoperative length of stay was defined as days of hospital stay in the acute care setting. All patients received PCA immediately after extubation and were maintained on it until they were transferred out of the surgical intensive care unit. Postoperative morphine usage was defined as milligrams of morphine per hour of use. None of the patients received continuous basal morphine infusion.

Data from categorical variables were summarized as frequencies (percentages), which were compared between groups by using the χ^2^ test or the Fisher exact test. Data from continuous variables were summarized as means ± standard deviations, which were compared between groups by using the Student *t* test. Statistical analyses were performed with SPSS version 17.0 (SPSS, Inc., Chicago, IL).

## Results

The 2 groups of patients did not differ significantly in terms of their preoperative comorbidities and demographics (Table 1). Patients who received xiphoid sternal plate reinforcement underwent more complex cardiac procedures and incurred longer myocardial ischemic times than patients who underwent standard wire closure. Also, the sternal-plate recipients used significantly less postoperative PCA morphine than the standard-closure patients (1.3 mg/h vs 3.6 mg/h, *P* = 0.008). No significant between-groups differences were noted for any of the other prespecified end points (Table 2).

**Table 1:** Preoperative characteristics

	Sternal plate (*n* = 8)	No sternal plate (*n* = 14)	*P* value
Age (y)	64 ± 5	61 ± 6	0.23
Gender (male)	8 (100.0)	14 (100.0)	1.00
Body mass index (kg/m^2^)	40.6 ± 4.2	38.6 ± 2.8	0.25
Diabetes mellitus	6 (75.0)	8 (57.1)	0.40
Peripheral vascular disease	0 (0)	1 (7.1)	0.44
Hypertension	8 (100.0)	14 (100.0)	1.00
Cerebral vascular disease	1 (12.5)	3 (21.4)	0.60
COPD	4 (50.0)	2 (14.3)	0.88
Current smoker	2 (25.0)	2 (14.3)	0.07
Creatinine level (mg/dL)	1.2 ± 0.2	1.1 ± 0.2	0.45
Albumin level (g/dL)	3.8 ± 0.4	3.6 ± 0.2	0.32
Ejection fraction (%)	50 ± 10	51 ± 8	0.79

Data presented as mean ± standard deviation or absolute number (percentage). *COPD* chronic obstructive pulmonary disease

**Table 2:** Intraoperative and outcome measures

	Sternal plate (*n* = 8)	No sternal plate (*n* = 14)	*P* value
Isolated CABG	3 (37.5)	13 (92.9)	0.005
Isolated AVR	1 (12.5)	1 (7.1)	0.67
Cardiopulmonary bypass time (min)	145 ± 57	98 ± 41	0.06
Myocardial ischemic time (min)	100 ± 46	58 ± 31	0.04
Time in OR (min)	362 ± 82	413 ± 60	0.15
Length of stay (d)	17 ± 5	11 ± 3	0.07
Morphine usage (mg/h)	1.3 ± 1.2	3.6 ± 2.3	0.008
Wound drainage	0 (0)	0 (0)	1.00
Sternal dehiscence	0 (0)	1 (7.1)	0.44
Mediastinitis	0 (0)	0 (0)	1.00
30-day mortality	0 (0)	0 (0)	1.00

Data presented as mean ± standard deviation or absolute number (percentage). *CABG* coronary artery bypass grafting, *AVR* aortic valve replacement, *OR* operating room

With the exception of 1 patient in the standard-closure group, all patients reported sternal stability and had no evidence of sternal dehiscence at their most recent follow-up clinic visit (a median of 27 months postoperatively; range, 8.4–49.3 months). There were no wound seromas or complications attributable to sternal closure in the sternal-plate patients.

**Fig. 2:**
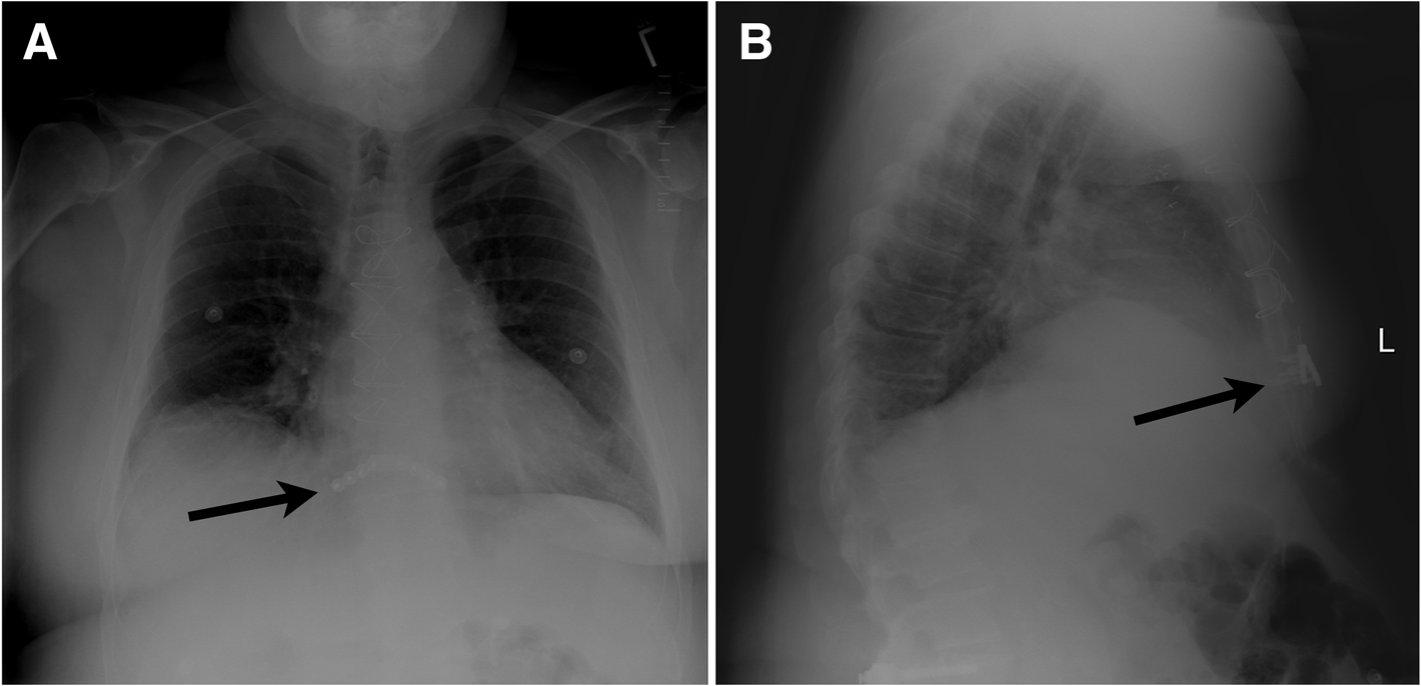
Plain chest radiographs taken 2 weeks after placement of a Synthes titanium plate (arrows). **a** Posterior-anterior view. **b** Lateral view

**Fig. 3:**
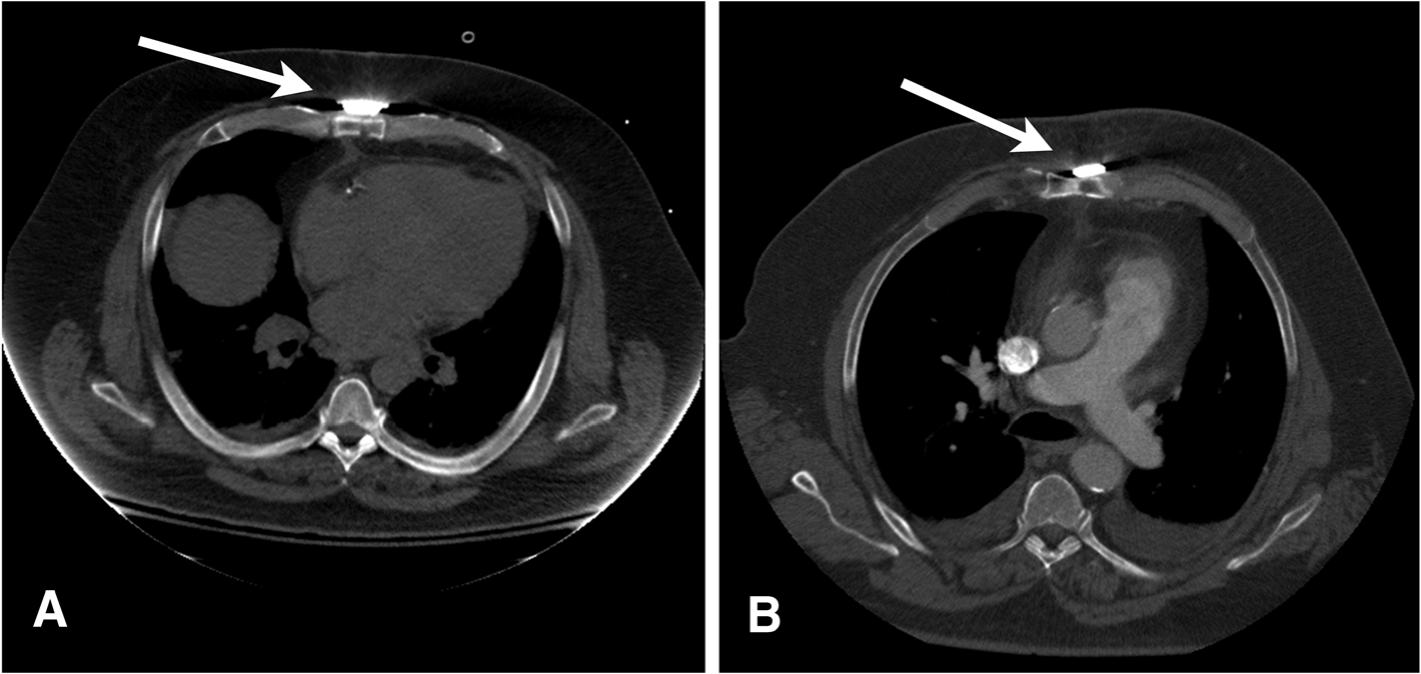
**a**. Chest computed tomography scans obtained from 2 different patients, taken 1 week (**a**) and 1 month (**b**) after placement of a sternal plate (arrows)

The approximate time needed for placement of single sternal plate reinforcement was 15 min. Routine postoperative plain chest radiography confirmed the position of the sternal plate (Fig. 2). Chest CT scans, obtained 1 week postoperatively to rule out early mediastinitis (Fig. 3a) and 1 month postoperatively to rule out pulmonary embolism (Fig. 3b), provided radiographic evidence of near-complete sternal union.

## Discussion

As the prevalence of obesity increases in our population, more morbidly obese patients are being referred for surgical myocardial revascularization [1, 2]. Morbid obesity is a well-known risk factor for sternal dehiscence and post-sternotomy mediastinitis [6]. Our group has previously published outcomes of transverse sternal plating in secondary sternal reconstruction [13]. The feasibility of sternal plating in primary closure after median sternotomy is not as well documented and is less frequently used by cardiac surgeons than secondary reconstruction, perhaps because of concerns about increasing the operative time or the risk of infection.

Although a few studies have explored the use of rigid sternal plate fixation for primary closure in patients deemed to be at high risk for sternal dehiscence, these studies included heterogeneous groups of patients whose sternal plate placements ranged from a single plate to full sternal plate fixation without wire-cerclage. These patients had an overall sternal dehiscence rate of 3 to 9% [16, 17]. Human cadaveric studies show that a single sternal plate placed on the xiphoid area along with traditional wire-cerclage has a similar lateral tensile strength to that of full sternal plating without wires [15]. Therefore, we hypothesized that reinforcing the xiphoid area with a single sternal plate in addition to the usual wire-cerclage is a feasible novel technique for primary sternal closure in morbidly obese patients.

In our study, we placed a single sternal plate in the xiphoid area in addition to performing a traditional wire closure. This technique achieved satisfactory primary sternal union without any increased incidence of infection or seroma formation in our 8 sternal-plate recipients, and the average plate implantation time was only 15 min. Our study demonstrated the feasibility of performing sternal plate reinforcement for primary closure without increasing the patient’s risk of infection or significantly extending operative time. Although no significant differences were seen in the majority of prespecified outcome measures between the sternal-plate group and the standard-closure group, the small sample size may not have given the study enough power to detect true differences in low-frequency outcomes, such as the incidence of sternal dehiscence.

Nevertheless, we were able to show a considerable reduction in the use of PCA morphine for immediate postoperative pain control in the sternal-plate recipients compared to the standard-closure patients. Even with the relatively small sample size of this pilot study, this difference was statistically significant (*P* = 0.008). Therefore, it is unlikely that this difference in narcotics usage is simply due to chance. This finding has major implications for patients. It potentially can lower the patient’s healthcare costs associated with narcotic usage and pain improvement. It can also potentially decrease complications of surgery related to narcotic usage, such as effects on mental status, constipation, and dependence on these medications.

Furthermore, this striking result may be attributable to improved early sternal stability in patients who received xiphoid sternal plate reinforcement in addition to standard wire-cerclage. Studies have shown that after primary sternal closure, the xiphoid area is the area of greatest mechanical stress [19–21]. This area is most prone to sternal nonunion and generally, is the site of surgical deep wound infections. In morbidly obese patients, the lateral stress is increased even more because of their body habitus. Morbidly obese patients have more soft tissue than their normal-weight counterparts, and this tissue places additional stress on the sternotomy closure, both laterally by the chest wall and inferiorly by the abdominal wall. As with any bone fracture, stress at the fracture site causes pain by stimulating pain fibers in the periosteum [22, 23]. Controlling this pain is the rationale for early immobilization by either open fixation or cast placement for most types of bony fractures. In our patients, it is possible that the xiphoid plate reinforcement stabilized a stressed sternotomy closure, thus increasing early sternal stability and reducing postoperative pain.

Sternal dehiscence has been associated with the development of post-sternotomy mediastinitis in morbidly obese patients [24]. All 8 patients who received xiphoid plate reinforcement had sternal stability at their last follow-up visit, whereas 1 patient in the standard-closure group developed sternal dehiscence. This finding is not surprising, because the plate enhances the sternal union at the lowermost aspect of the sternotomy closure. The patients’ chest CT scans (Fig. 3) showed near-perfect sternal approximation as early 1 week and 1 month postoperatively. The sternal plate undoubtedly has much more tensile strength than traditional stainless steel wires, as evidenced by biomechanical studies [9, 25], so it may be better able to resist the additional stresses placed upon it by a morbidly obese patient’s body habitus. Therefore, single sternal plate xiphoid reinforcement has the potential to reduce sternal dehiscence, which may decrease the patient’s risk of the dreaded complication of post-sternotomy mediastinitis [24].

Our study is subject to the limitations inherent in a retrospective review. There was obvious selection bias regarding whether a patient received sternal plate reinforcement, because this technique was used only in morbidly obese patients. We attempted to control for this bias by comparing the sternal plate recipients’ results to those of patients with a similar risk profile who underwent standard wire closure performed by the same surgeon. Also, because the VA patient population is limited to veterans, all patients were male. Consequently, we cannot generalize our results to female patients. Lastly, because of the small number of patients included in our study, our ability to examine low-frequency outcomes such as mediastinitis and sternal dehiscence was limited. Nonetheless, this study provides pilot data for future randomized control trials involving more patients. Furthermore, a single sternal plate at the xyphoid may make salvage re-sternotomy in a non-operative setting difficult. In this setting, large shears can cut through either side of the cartilaginous portions of the ribs. Also, there is a plate cutter that can be used to split the titanium plate in the middle. Specifically, the sternal plates used in our cohort has a pin which connects 2 sides. This pin may simply be disconnected with a small clamp.

## Conclusion

In conclusion, our results suggest that a single sternal plate xiphoid reinforcement for primary closure in morbidly obese patients is a feasible option that achieves excellent sternal union, provides sternal stability, and may reduce postoperative narcotics use. Further studies are needed to determine the potential benefits of single sternal plate xiphoid reinforcement in morbidly obese patients, who are at high risk of developing sternal dehiscence.

## Abbreviations

BMI: Body mass index
CABG: Coronary artery bypass grafting
CAD: Coronary artery disease
CICSP: Continuous Improvement in Cardiac Surgery Program
CT: Computed tomography
DSWI: Deep sternal wound infections
MEDVAMC: Michael E. DeBakey Veterans Affairs Medical Center
PCA: Patient-controlled analgesia
VA: Veterans Affairs
